# Comparing Effectiveness Between a Mobile App Program and Traditional Cognitive Behavior Therapy in Obsessive-Compulsive Disorder: Evaluation Study

**DOI:** 10.2196/23778

**Published:** 2021-01-19

**Authors:** Hyunchan Hwang, Sujin Bae, Ji Sun Hong, Doug Hyun Han

**Affiliations:** 1 Department of Psychiatry Chung-Ang University Hospital Seoul Republic of Korea; 2 Office of Research Chung-Ang University Seoul Republic of Korea

**Keywords:** obsessive-compulsive disorder, exposure and response prevention, cognitive behavior therapy, cortico-striato-thalamo-cortical tract, functional connectivity, prevention, cognitive, mental illness, behavior therapy

## Abstract

**Background:**

This study proposes a digital program for the treatment of mental illness that could increase motivation and improve learning outcomes for patients. Several studies have already applied this method by using an exposure and response prevention–inspired serious game to treat patients with obsessive-compulsive disorder (OCD).

**Objective:**

We hypothesized that a mobile cognitive behavior therapy (CBT) program would be as effective in treating OCD as traditional offline CBT. In addition, the treatment efficacy in response to mobile CBT for OCD might be associated with increased brain activity within the cortico-striato-thalamo-cortical (CSTC) tract.

**Methods:**

The digital CBT treatment program for OCD, OCfree, consists of 6 education sessions, 10 quests, and 7 casual games. Information was gathered from 27 patients with OCD (15 offline CBT and 12 OCfree CBT). During the 6-week intervention period, changes in clinical symptoms and brain function activity were analyzed.

**Results:**

There was no significant difference in the change in OCD symptoms and depressive symptoms between the two groups. However, the OCfree group showed greater improvement in anxiety symptoms compared to the offline CBT group. Both offline CBT and OCfree CBT increased the functional connectivity within the CSTC tract in all patients with OCD. However, CBT using OCfree showed greater changes in brain connectivity within the thalamus and insula, compared to offline CBT.

**Conclusions:**

OCfree, an OCD treatment app program, was effective in the treatment of drug-naïve patients with OCD. The treatment effects of OCfree are associated with increased brain connectivity within the CSTC tract. Multisensory stimulation by education, quests, and games in OCfree increases the activity within the thalamus and insula in patients with OCD.

## Introduction

### Overview

Obsessive-compulsive disorder (OCD) is a debilitating mental disorder associated with significant social and occupational impairments [1], affecting 2%-3% of the population worldwide [2]. It is diagnosed by the presence of obsessions, compulsions, or both [2]. Obsessions are characterized as intrusive thoughts or images that are often unwanted and repeat constantly. Compulsions, on the other hand, are defined as repetitive behaviors or mental thinking that people feel they need to do, often to counter the obsessions.

Along with obsession and compulsion, functional brain changes in patients with OCD have also been noted. Many studies have found the cortico-striato-thalamo-cortical (CSTC) tract to be one of the crucial brain circuits involved in OCD [3,4]. Within the CSTC tract, patients with OCD showed different brain activity in response to various stimuli [5,6]. In response to emotion-related tasks, patients with OCD showed overactivation within the anterior cingulate cortex, insula, caudate head, and putamen, which are thought to play a part in salience, arousal, and habit responding [5]. In addition, underactivation was shown in the medial prefrontal cortex and posterior caudate, which are associated with cognitive and behavioral control [5]. Regions outside the CSTC tract are also of interest, as Zhang et al showed altered functional connectivity (FC) in resting-state functional magnetic resonance imaging (Rs-fMRI) between the cerebellum and CSTC circuit in OCD patients [6].

Of several treatment options for OCD, cognitive behavior therapy (CBT) with exposure and response prevention (ERP) is regarded as one of the first choices in many clinical guidelines [7,8]. The National Institute for Health and Care Excellence recommends intensive CBT (over 10 therapist hours including ERP) or a selective serotonin reuptake inhibitor (SSRI) as initial treatment [7]. The American Psychiatric Association also recommends CBT with ERP, SSRIs, or both as first-line treatments [8]. Recent reviews on the literature have found that CBT with ERP has larger effect sizes than pharmacotherapy, although interpreting this as CBT with ERP being better than medication must be avoided as there are many factors to consider [9].

However, CBT with ERP is not without its flaws. One of the main disadvantages of CBT is that it is time consuming, and efficacy is tied to the participant’s engagement with the therapy [10]. Moreover, Barnes et al found that homework, one of the key elements of CBT, was one of the main reasons for low adherence because it is linked to negative school homework experiences [10].

Many methods have been devised to overcome this disadvantage. The delivery of mental health services through the internet is one method being considered [11,12]. This method has the potential to increase patient engagement and availability [11]. In a randomized controlled trial of family-based treatment for OCD, internet delivered cases showed higher response rates than clinic cases, and this difference persisted after treatment, although the difference was not statistically significant [12]. Another method used is serious games. Eichenberg and Schott argued that serious games could increase motivation and improve learning outcomes, thereby supplementing some of the weaknesses of the conventional internet-mediated health program [13].

Serious games are defined as games developed with a purpose other than entertainment [14]. Notable examples of these are games designed to improve aircrew training [15] or education [16]. Serious games are also used in the medical field to help patients. A meta-analysis and systematic review showed that serious games for mental health were effective in reducing symptoms related to depression, autism spectrum disorder, post-traumatic stress disorder, attention-deficit/hyperactivity disorder, and alcohol use disorder [14]. Serious games have also been shown to lower anxiety and related symptoms. Kim et al reported that a serious game helped lower depression and anxiety in breast cancer patients [17]. A pilot study also showed that a serious game helped reduce anxiety and pain in children before day-care surgery [18]. More recently, Hong et al used an ERP-inspired serious game to treat OCD patients; the patients showed improved OCD symptoms, which correlated with increased brain connectivity between the dorsal anterior cingulate cortex and the prefrontal cortex, after 3 weeks of game play [19].

### Hypothesis

To our knowledge, a mobile app using serious games with both ERP and CBT for OCD has not yet been developed. Therefore, we designed a mobile app based on these theories and conducted a randomized controlled trial comparing the developed program directly with traditional CBT with ERP in OCD patients. We hypothesized that the mobile CBT program would be as effective in the treatment of OCD as traditional offline CBT. In addition, the treatment efficacy in response to mobile CBT for OCD might be associated with increased brain activity within the CSTC tract.

## Methods

### Participants

Through advertisements for treatment of OCD, 32 patients with OCD were recruited from the Department of Psychiatry at Chung-Ang University Hospital. All patients with obsession or compulsion symptoms were screened using the structured Clinical Interview for the Diagnostic and Statistical Manual of Mental Disorders, Fifth Edition (DSM-5), and diagnosed by a psychiatrist (DHH). Inclusion criteria were as follows: (1) age>18 years, (2) diagnosed with OCD based on DSM-5, (3) drug naïve, and (4) right-handedness. Exclusion criteria were as follows: (1) IQ<80; (2) history of medical or other psychiatric disorders; (3) history of substance use disorders; (4) contraindications to MRI scanning, including claustrophobia or metal implant; and (5) current psychotherapy or medication treatment.

Of the 32 patients with OCD, 1 patient was excluded due to psychotic symptoms of hallucinations. The remaining 31 patients were randomly classified into two groups: an offline CBT group (n=16) and a web-based CBT using OCfree group (n=15). One patient in the offline CBT group and 1 patient in the OCfree group were excluded due to taking medication for anxiety reduction, and another patient in the OCfree group was excluded due to a brain infarction finding in the baseline fMRI. In the OCfree group, 1 patient did not complete the study protocol because they did not want to visit the hospital during the COVID-19 pandemic. Finally, the information of 27 patients with OCD (15 offline CBT and 12 OCfree CBT) was analyzed. Participants who entered the trial received a maximum of 100,000 won (around US $89) during the whole trial to compensate for travel fees. The institutional review board of Chung-Ang University Hospital approved this study, and all participants provided written informed consent.

### Study Design

A randomized and treatment-as-usual controlled design was applied for this study. Individual in-person CBT was selected as treatment-as-usual with reference to the American Psychiatric Association practice guidelines [20]. The guideline recommends CBT or medication (SSRI) as the first-line treatment for OCD, states that individual and group CBT seem equally effective, and mentions that internet-delivered CBT is promising and deserving of further research [20]. After screening, all patients with OCD were randomly assigned to receive offline CBT once per week or web-based CBT using OCfree daily for 6 weeks, according to the randomization sequence generated using SPSS version 24.0 (IBM Corp, Armonk, NY, USA), with a 1:1 allocation (offline CBT:CBT using OCfree). At baseline and after intervention, all patients with OCD were assessed with the Korean version of the Yale-Brown Obsessive Compulsive Scale (Y-BOCS) for OCD symptoms [21,22], the Korean version of the Beck Depression Inventory-II (BDI) for depressive symptoms [23,24], and the Korean version of the Beck Anxiety Inventory (BAI) for anxiety symptoms [25,26].

After baseline psychological scales and fMRI data were acquired, the offline CBT group had an hour-long individual session per week with a psychiatrist for 6 weeks. The CBT sessions were designed similarly to the traditional 10-session CBTs but shortened to match the online CBT session numbers. Homework was given and checked for each session.

The OCfree CBT group also had an individual psychiatrist assigned to meet the participant each week and oversee the CBT for 6 weeks. The psychiatrist in the OCfree group used the OCfree program to conduct CBT, and each session lasted approximately 40 minutes. The program has a scheduler system that assigns participants different programs within OCfree for their daily use, and the participant can use different parts of the program as many times as they wish. The psychiatrist would check the program each week for compliance.

### OCfree Program

**Figure 1:**
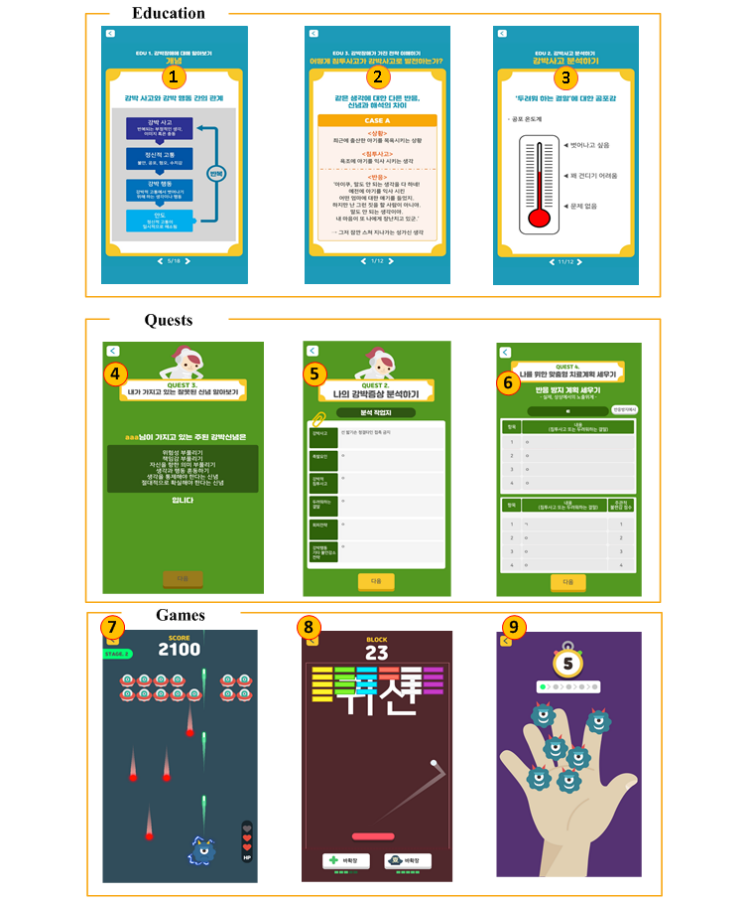
OCfree program: (1) learning about OCD: learn about symptoms, causes, and treatments, (2) analyzing obsessions: trigger factors for obsession and compulsive behaviors, compulsive infiltration, fearful ending, (3) understanding strategies with OCD, (4) understanding false beliefs related to obsession, (5) analyzing the symptoms of obsessions and compulsions with 8 panels, (6) creating a customized treatment plan for you, (7) shooting game, (8) break block game, (9) germ-removing game.

The mobile app (OCfree) for obsessive-compulsive disorder treatment consists of three categories: education, quests, and serious games. The education category consisted of 6 sessions: (1) learning about OCD: learn about symptoms, causes, and treatments; (2) analyzing obsessions: trigger factors for obsession and compulsive behaviors, compulsive infiltration, fearful ending; (3) understanding strategies with OCD; (4) customized treatment plan; (5) factors for change: a firm resolution; and (6) explanation of cognitive therapy. The learning time of each education session was 20-30 minutes. The quest category consists of 10 subcategories. Those are paired with each education session and supplied to patients as homework: (1) assessment of obsession and compulsion using 65 questions for obsession and 65 questions for compulsion, (2) analyzing the symptoms of obsession and compulsion with 8 panels, (3) understanding false beliefs related to obsession, (4) creating a customized treatment plan for you, (5) preparing for change by yourself, (6) working book for cognitive therapy and 4 adjuvant categories, (7) practice postponing anxiety, (8) ERP using imagination via voice recording, (9) identifying emotions, and (10) practicing choosing. The game category consists of 7 casual games: (1) shooting game, (2) break block game, (3) germ-removing game, (4) doubting and checking game, (5) symmetry and ordering game, (6) numbering and counting game, and (7) mental ritual game (Figure 1).

The shooting game is similar to the fixed shooter arcade game, Galaga. By controlling a spaceship, the players can destroy aliens while avoiding enemies’ projectiles. The spaceship is displaced with the object that OCD patients dislike (want to avoid) such as a needle, knife, death, germ, or airplane. The break block game is a modified version of the classic Breakout block game. The players use the paddle to bounce the ball and destroy the bricks. Behind the brick, there is a “word” that is associated with the obsession. When the bricks are completely destroyed, the “word” disappears. Before starting the block game, the OCD patients type the “word” that is associated with the obsession. In the germ-removing game, players can remove pictorial germs represented on the palm of a hand until only one germ remains. The OCD patient should wait 5 seconds before removing the last one. The number of germs increases when the stage is cleared. In the doubting and checking game, players can touch a pictorial faucet on a screen until the number “1” is represented. Like the germ-removing game, OCD patients should wait 5 seconds before the last touch (Figure 1). The objects of the checking can change in accordance with the patients’ obsessions, including a doorknob and gas valve. In the symmetry and ordering game, players can place books on a bookshelf in accordance with size and color. OCD patients should wait 5 seconds before arranging the last one. More books of various sizes and colors are presented after each stage is cleared. In the numbering and counting game, players type a number that they are preoccupied with. This number is the same as the number of eggs on the screen. The players touch the eggs to hatch them until the last egg remains. The OCD patients should wait 5 seconds before touching the last egg to hatch. In the mental ritual game, players type a word that they are preoccupied with. The word then multiplies and spreads on the screen. Of the multiplied words, 10% are modified in a different or wrong spelling (one or two characteristics are different), compared to the original spelling. OCD patients should only touch words written in the original spelling.

### Brain Imaging Data Acquisition and Processing

A 3.0 Tesla Philips Achieva scanner was used to acquire Rs-MRIs. Only right-handed participants entered the trial due to reports showing functional and anatomical differences in the brain between right- and left-handedness [27]. A total of 230 volumes for 720 seconds were gathered using the following parameters: repetition time/echo time=3000/40 milliseconds, 40 slices, 64×64 matrix, 90° flip angle, 230-mm field of view, and 3-mm section thickness, without a gap. Using the programs of the Data Processing Assistant for Rs-fMRI [28] and the Rs-fMRI Data Analysis Toolkit (REST) [29], all acquired imaging data were prepared for preprocessing and processing. Brain activity within regions of interest (ROIs) was derived from the fractional amplitude of low-frequency fluctuations (fALFF), extracted using REST software. Seed-based FC analysis was performed using the seed ROI extracted from the previous step of correlation comparison between Y-BOCS and fALFF. More details of the fMRI data preprocessing and processing were described in our previous study [30].

### Statistical Analysis

Demographic and clinical characteristics of the offline and OCfree groups were analyzed using the Mann-Whitney U test. The differences in sex ratio and symptom improvement between the two groups were analyzed using a chi-square test. Symptom improvement was defined as a decrease of 2.85 in 25% of Y-BOCS scores based on a reliable change index of symptoms (standard error of measure=1.03, effect size=0.91) [31]. Statistical significance was set at *P*<.05.

The correlation between the fALFF and Y-BOCS scales was calculated using multiple regression analysis in Statistical Parametric Mapping 12 (SPM12; Wellcome Centre for Human Neuroimaging). The changes in fALFF from baseline to 4 weeks in all patients were estimated using a paired *t* test in SPM12. The differences in the change of fALFF and FC from the thalamus to other brain areas between the offline and OCfree groups were estimated using repeated-measures analysis of variance in SPM12. The ROIs were extracted based on the cluster in the t-map with a defined threshold (uncorrected *P*<.001, voxels>20).

## Results

### Demographic Characteristics and Clinical Scales

There were no significant differences in age, years of education, Y-BOCS scores, BDI scores, and BAI scores between the offline CBT and OCfree groups at baseline (Table 1).

**Table 1:** Demographic data and clinical scales.

Characteristic		OCfree group (n=12)	Offline CBT^a^ group (n=15)
Age (years), mean (SD)		25.7 (7.7)	24.7 (10.7)
**Sex, n**			
	Male	5	6
	Female	7	9
Education (years), mean (SD)		13.3 (1.8)	13.8 (2.1)
**Economic status (income)^b^, n**			
	Low	2	3
	Middle	7	9
	High	3	3
**Y-BOCS^c^, mean (SD)**			
	Pretreatment	21.9 (5.7)	19.5 (4.1)
	Posttreatment	16.7 (5.4)	15.9 (4.7)
**BDI^d^, mean (SD)**			
	Pretreatment	19.3 (4.9)	16.5 (11.2)
	Posttreatment	9.3 (5.3)	10.3 (9.5)
**BAI^e^, mean (SD)**			
	Pretreatment	19.8 (10.7)	16.2 (11.7)
	Posttreatment	9.4 (8.6)	11.0 (9.4)

^a^CBT: cognitive behavior therapy.

^b^Economic status (income): low, <US $20,000/year; middle, US $20,000-40,000/year; high, >US $40,000/year.

^c^Y-BOCS: Yale-Brown Obsessive Compulsive Scale.

^d^BDI: Beck Depression Inventory.

^e^BAI: Beck Anxiety Inventory.

**Figure 2:**
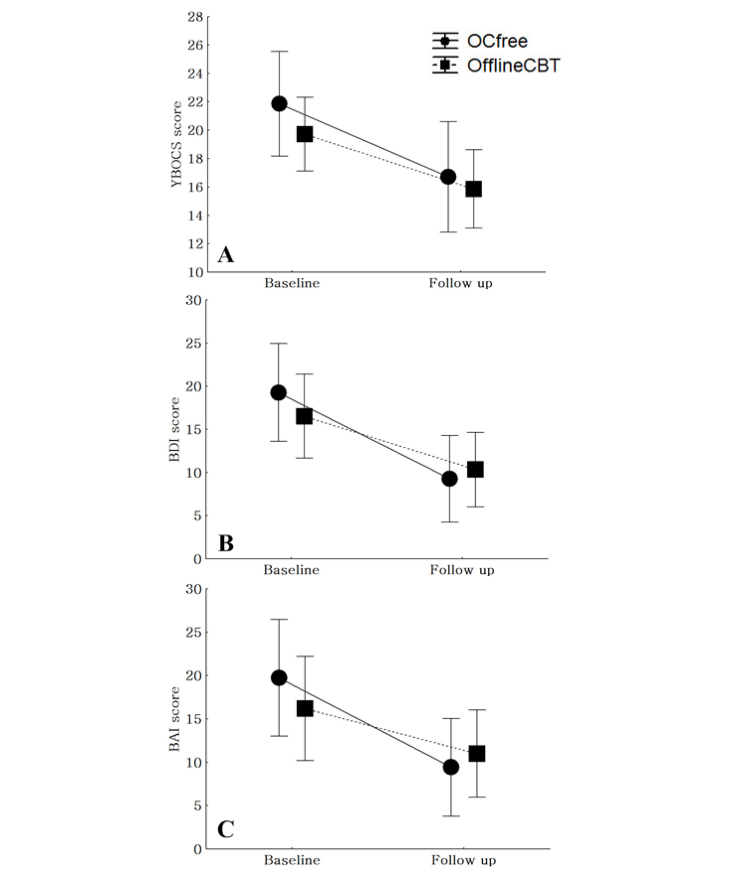
Comparisons of the changes of (A) Y-BOCS, (B) BDI, and (C) BAI scores between the offline CBT group and OCfree group. BAI: Beck Anxiety Inventory. BDI: Beck Depression Inventory. CBT: cognitive behavior therapy. Y-BOCS: Yale-Brown Obsessive Compulsive Scale.

The number of improved OCD patients in the OCfree group (improvement vs nonimprovement: 8/12, 67% vs 4/12, 33%) was greater than that observed in the offline group (8/15, 53% vs 7/15, 47%), but the difference was not statistically significant (*χ*^2^=0.5; *P*=.69). There were also no significant differences in the change of Y-BOCS scores (*F*=0.50; *P*=.48) and BDI scores (*F*=2.16; *P*=.16) between the two groups. Compared to the offline CBT group, the OCfree group showed greater improvement in BAI scores (*F*=5.74; *P*=.02) (Figure 2).

### Results of the Program Usage and Satisfaction Survey

On average, program compliance was 91.4%. The 6 education modules were used 6.0 times each, and the 10 quest modules were used 10.8 times each during the 6 weeks of program usage. The 7 game modules were used, on average, 12.1 times each.

An anonymous survey was taken at the end of the program, and 10 out of 12 participants completed it. The participants were given, among other things, a choice of 1 to 5 stars to measure overall satisfaction, 1 being the lowest and 5 being the highest. The overall satisfaction was 3.4 stars out of 5. The majority of 2- or 3-star ratings were due to minor errors in the program, giving it a somewhat crude feeling. The 4- and 5-star reviews stated that the program helped them get to know their obsessions and compulsions better. Of the 10 completed surveys, 5 (50%) said that they would like to continue using the program even after the trial, and 7 (70%) wished to recommend it to other people with similar symptoms. There have been no reports of adverse effects of the program. Adverse effects were checked by the psychiatrist conducting the CBT sessions each week and not asked about in the anonymous survey.

### Correlation Between the Y-BOCS Scale and Brain Activity (fALFF)

**Figure 3:**
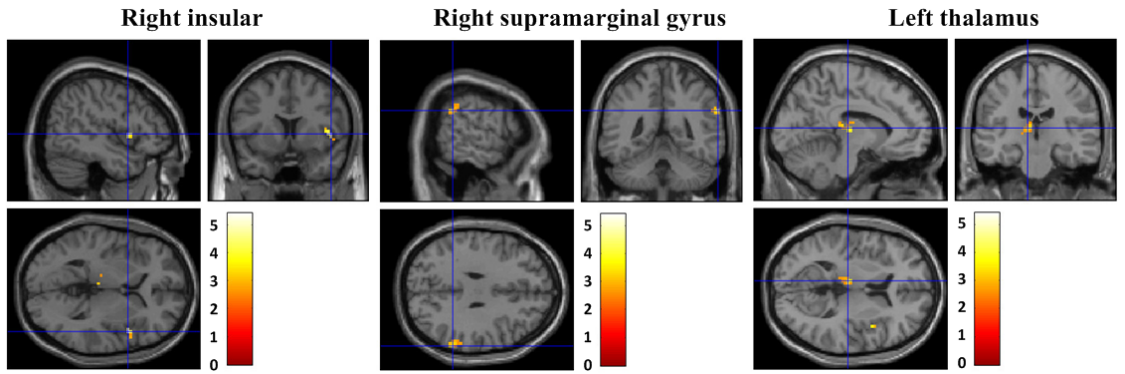
Correlation between the Yale-Brown Obsessive Compulsive Scale and brain activity.

In all patients with OCD, Y-BOCS scale scores were negatively correlated with fALFF within the right insular (Talairach code x, y, z: 48, 9, 3; T=5.41; *P*_uncorrected_<0.001; k_E_=20, Brodmann area [BA] 13), right parietal supramarginal gyrus (x, y, z: 63, −45, 30; T=4.42; *P*_uncorrected_<0.001; k_E_=26, BA 40), and left thalamus (x, y, z: −9, −21, 9; T=4.23; *P*_uncorrected_<0.001; k_E_=29) (Figure 3).

### Comparison of the Changes in fALFF Between the OCfree Group and Offline CBT Group

**Figure 4:**
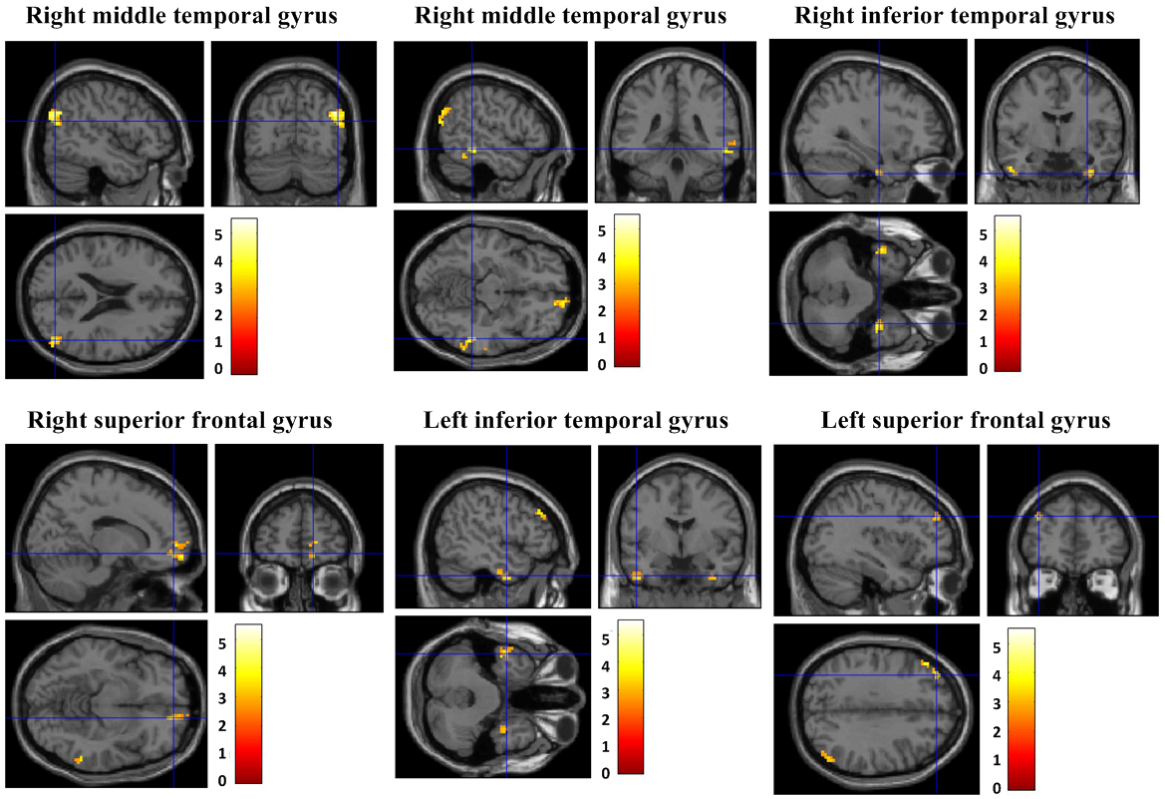
Comparison of the changes in fractional amplitude of low-frequency fluctuations between the OCfree and offline cognitive behavior therapy groups.

During the intervention period, the fALFF within the right middle temporal gyrus (x, y, z: 48, −72, 21; T=5.50; *P*_uncorrected_<0.001; k_E_=101, BA 39), right middle temporal gyrus (x, y, z: 54, −39, −12; T=4.41; *P*_uncorrected_<0.001; k_E_=73, BA 20), right inferior temporal gyrus (x, y, z: 33, −6, −39; T=4.30; *P*_uncorrected_<0.001; k_E_=216, BA 20), right superior frontal gyrus (x, y, z: 15, 54, −9; T=4.24; *P*_uncorrected_<0.001; k_E_=34, BA 10), left inferior temporal gyrus (x, y, z: −48, −3, −36; T=4.22; *P*_uncorrected_<0.001; k_E_=39, BA 20), and left superior frontal gyrus (x, y, z: −36, 45, 33; T=4.02; *P*_uncorrected_<0.001; k_E_=31, BA 9) had increased in all patients with OCD (Figure 4).

During the intervention period, the OCfree group showed increased fALFF within the right parahippocampal gyrus (x, y, z: 39, −42, −6; T=4.60; *P*_uncorrected_<0.001; k_E_=35, BA 19), compared to the offline CBT group.

### Comparison of the Changes in FC Between the OCfree Group and Offline CBT Group

**Figure 5:**
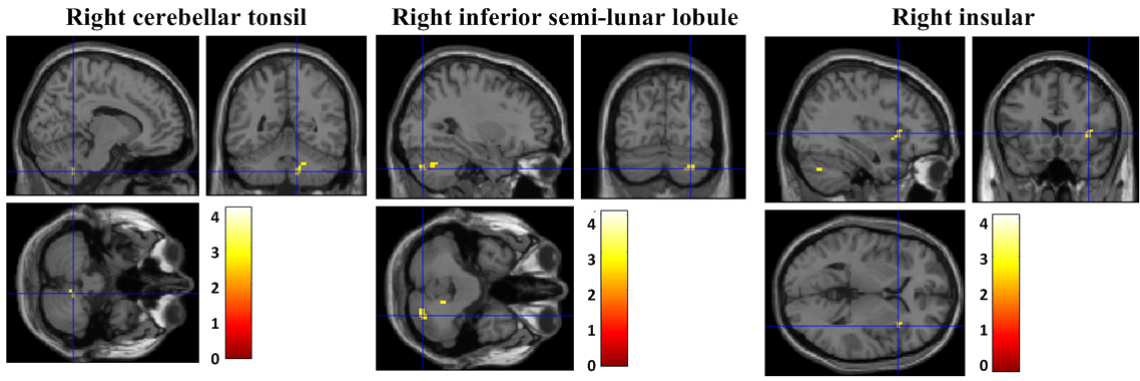
The changes in functional connectivity in all the patient groups (OCfree and offline cognitive behavior therapy group).

During the intervention period, the FC from the left thalamus to the right cerebellar tonsil (x, y, z: 12, −51, −45; T=3.18; *P*_uncorrected_<0.001; k_E_=24), right cerebellar inferior semilunar lobule (x, y, z: 30, −75, −39; T=3.08; *P*_uncorrected_<0.001; k_E_=26), and right insular (x, y, z: 36, 18, 3; T=3.01; *P*_uncorrected_<0.001; k_E_=21) had increased in all patients with OCD (Figure 5).

**Figure 6:**
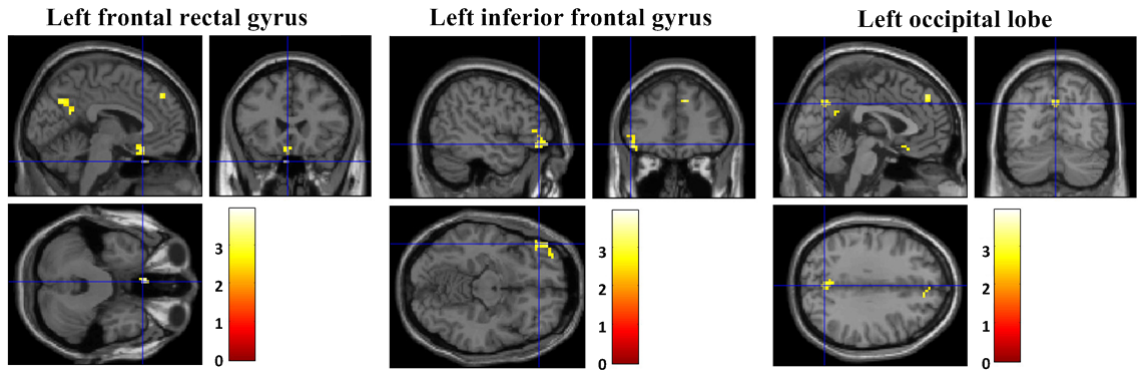
Changes in functional connectivity in the offline cognitive behavior therapy group.

During the intervention period, the FC from the left thalamus to the left frontal rectal gyrus (x, y, z: −3, 24, −33; T=3.51; *P*_uncorrected_<0.001; k_E_=30), left inferior frontal gyrus (x, y, z: −48, 39, −12; T=3.39; *P*_uncorrected_<0.001; k_E_=53, BA 47), and left occipital lobe (x, y, z: 0, −69, 33; T=3.25; *P*_uncorrected_<0.001; k_E_=39, BA 7) was increased in the offline CBT group (Figure 6).

**Figure 7:**
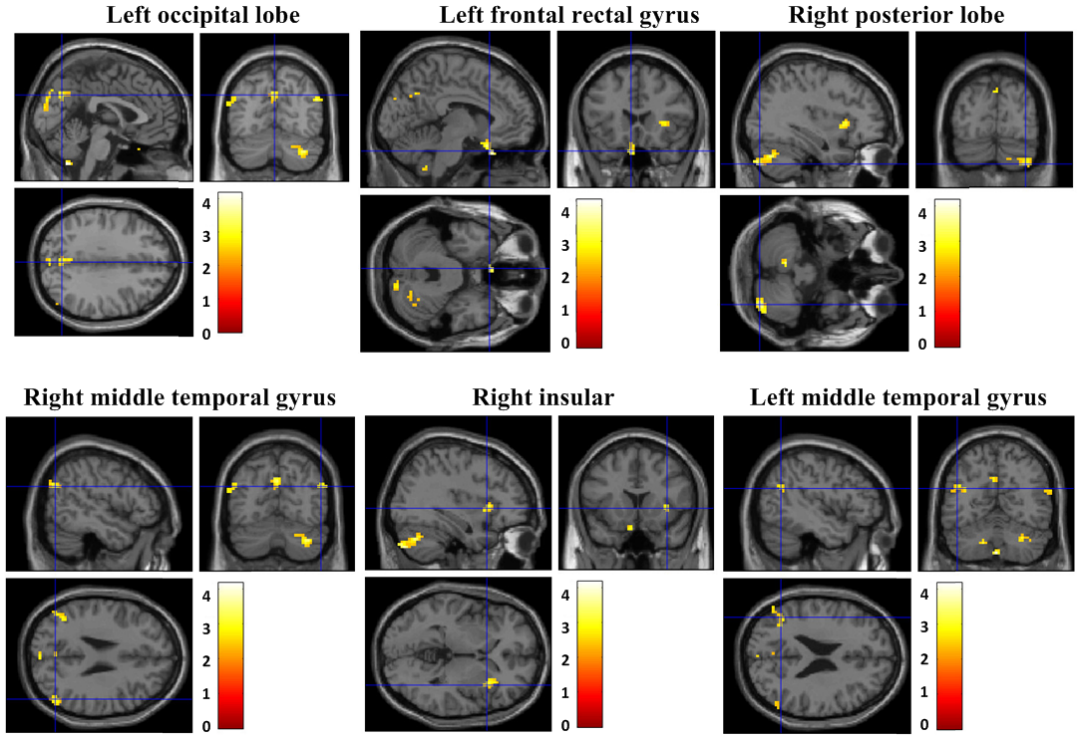
Changes in functional connectivity in the OCfree group.

During the intervention period, the FC from the left thalamus to the left occipital lobe (x, y, z: 0, −63, −51; T=4.18; *P*_uncorrected_<0.001; k_E_=47, BA 7), left frontal rectal gyrus (x, y, z: −6, 21, −30; T=3.95; *P*_uncorrected_<0.001; k_E_=30, BA 11), right cerebellar posterior lobe (x, y, z: 36, −81, −45; T=3.71; *P*_uncorrected_<0.001; k_E_=172, BA 47), right middle temporal gyrus (x, y, z: 51, −69, 27; T=3.05; *P*_uncorrected_<0.001; k_E_=35, BA 39), right insular (x, y, z: 36, 15, 0; T=3.02; *P*_uncorrected_<0.001; k_E_=31), and left middle temporal gyrus (x, y, z: −45, −60, 24; T=3.01; *P*_uncorrected_<0.001; k_E_=39, BA 39) had increased in the OCfree group (Figure 7).

During the intervention period, the OCfree group showed an increase in FC from the left thalamus to the left insular (x, y, z: −30, −39, 15; T=3.51; *P*_uncorrected_<0.001; k_E_=34, BA 13), compared to the offline CBT group.

## Discussion

### Principal Findings

OCfree, an OCD treatment app program, was as effective at improving OCD symptoms as offline CBT for OCD. The severity of OCD assessed with the Y-BOCS scale in all patients was negatively associated with brain activity within the emotion perception network, including the thalamus and insular. Both offline CBT and OCfree CBT improved OCD symptoms and increased FC within the CSTC tract in all patients with OCD. However, CBT using OCfree showed greater changes in fALFF within the thalamus and insular, compared to offline CBT.

### The Effectiveness of OCfree, an OCD Treatment Program, on the Improvement of OCD Symptoms

The OCfree program was as effective as offline CBT for the improvement of OCD symptoms in OCD patients. Overall compliance with OCfree was 91.4% and overall satisfaction was rated 3.4 out of 5 stars. The internet delivery CBT system for OCD has already been reported to be as effective as offline CBT [12]. In our previous study, an ERP-inspired serious game for OCD improved symptoms in OCD patients [19]. Moreover, OCfree greatly improved anxiety compared to offline CBT. We believe that the OCfree web-based delivery system, including education and quests, may enable easy patient access to the treatment system, and serious games in OCfree may increase interest in treatment, as immersion is thought to be one of the merits of serious games. Easy access and frequent contact with patients in OCD management decrease patient anxiety [32-34]. In mood disorders, internet-assisted cognitive behavioral therapy is becoming an evidence-based cognitive treatment [35]. Serious game−assisted clinical treatments already suggest that serious games can increase affinity and treatment compliance in many areas, including cancer [36], obesity [37], and autism spectrum disorders [38].

### Comparison of the Changes in fALFF Between the OCfree and Offline CBT Group

Comparisons before and after the treatment period in this study showed increased brain activity within the frontal and temporal lobes in all OCD patient groups. Compared to the offline CBT group, the OCfree group showed increased brain activity within the right parahippocampal gyrus. A deficit in emotional perception was reported in patients with OCD [39]. Due to this deficit, repetitive meaningless thoughts occurred in patients with OCD [39]. In several fMRI studies of OCD patients, decreased brain activity within the frontal and temporal lobes has been reported [40,41]. Chen et al reported that patients with OCD showed decreased brain activity within the left medial prefrontal cortex, compared to healthy subjects [42]. In patients with obsessive and compulsive symptoms due to temporal lobe infarction, SSRI treatment would improve the obsessive and compulsive symptoms [43]. The parahippocampal gyrus is known to play a crucial role in the perception of emotion [44]. Within the retrosplenial and posterior cingulate gyri, the parahippocampal gyrus is thought to play a crucial role in facial expression recognition [44]. Taken together, we believe that the OCfree program would improve OCD symptoms in patients with OCD. Moreover, the improvement may be due to the increased brain activity within the brain regions associated with emotional perception.

### Comparison of the Changes in FC Between the OCfree and Offline CBT Group

The brain’s FC within the CSTC tract was increased in all patient groups. In several studies, altered (disconnected) brain FC within the CSTC tract in OCD patients has already been reported [6,45]. Based on these results, we suggest that both OCfree and offline CBT may present similar treatment mechanisms of increased FC within the CSTC tract.

Interestingly, compared to the offline CBT group, the OCfree group showed increased brain FC from the thalamus and insular. This result may be associated with various and multiple sources of sensory stimulation via education, quests, and games during play. The thalamus is thought to act as a hub that receives sensory signals from every sensory system and sends them to the associated cortex [46]. The insular functions are associated with sensorimotor processing, socioemotional processing, and cognitive functions [47]. Decreased brain activity within the thalamus [48] and insular [49] have been reported in patients with OCD. Considered together, OCfree with various stimulation systems may increase the thalamus and insular activity, compared to offline CBT. Although there was no difference in the efficacy of treatment between OCfree and offline CBT, future studies with a larger number of subjects may show greater efficacy in symptom improvement with OCfree, compared to offline CBT.

### Limitations

There were several limitations to this study. First, the small sample size and short-term intervention period were not sufficient for generalizing the results. Second, due to the exclusion criteria for medication use, OCD patients with severe anxiety symptoms were excluded from this study. These exceptions can affect the results of the anxiety comparison between the two groups. In addition, although we could not find any individual with co-occurrence of OCD and claustrophobia, lifetime comorbidity between OCD and specific phobias has been reported to be 22% [50]. Data from a Mexican mental health survey showed that 24.8% of adolescents with a specific phobia were afraid of closed spaces [51]. For the results of this OCD brain study, OCD patients who experience anxiety in closed spaces were excluded. For this reason, readers should be cautious about generalizing current results. Finally, this study did not recruit a true control group without any formal structured CBT due to ethical limitations. Future studies should recruit a larger number of participants and consider comorbid conditions, including anxiety and mood fluctuations, with a truer control group.

### Conclusions

OCfree, an OCD treatment program, was effective in the treatment of drug-naïve patients with OCD. The treatment effects of OCfree are associated with increased brain connectivity within the CSTC tract. Multisensory stimulation by education, quests, and games in OCfree increased activity within the thalamus and insular regions in patients with OCD.

## Abbreviations

BA: Brodmann area
BAI: Beck Anxiety Inventory
BDI: Beck Depression Inventory
CBT: cognitive behavior therapy
CSTC: cortico-striato-thalamo-cortical
DSM-5: Diagnostic and Statistical Manual of Mental Disorders, Fifth Edition
ERP: exposure and response prevention
fALFF: fractional amplitude of low-frequency fluctuations
OCD: obsessive-compulsive disorder
REST: Resting-state functional magnetic resonance imaging Data Analysis Toolkit
ROI: region of interest
Rs-fMRI: resting-state functional magnetic resonance imaging
SPM12: Statistical Parametric Mapping 12
SSRI: selective serotonin reuptake inhibitor
Y-BOCS: Yale-Brown Obsessive Compulsive Scale
